# Vitrification of canine ovarian tissues with polyvinylpyrrolidone preserves the survival and developmental capacity of primordial follicles

**DOI:** 10.1038/s41598-019-40711-6

**Published:** 2019-03-08

**Authors:** Mayako Fujihara, Takehito Kaneko, Miho Inoue-Murayama

**Affiliations:** 1 Wildlife Research Center, Kyoto University, Kyoto, 606-8203 Japan; 2 Division of Science and Engineering, Graduate School of Arts and Science, Iwate University, Morioka, 020-8551 Japan; 3 Department of Chemistry and Biological Sciences, Faculty of Science and Engineering, Iwate University, Morioka, 020-8551 Japan; 4 Wildlife Genome Collaborative Research Group, National Institute for Environmental Studies, Tsukuba, 305-8506 Japan

## Abstract

Ovarian tissue cryopreservation combined with immature follicle development can preserve female fertility in wildlife, regardless of age or reproductive timing. To investigate the effects of different cryopreservation methods and cryoprotectants on follicular survival and developmental capacity, ovarian cortical pieces from 15 dogs were cryopreserved by slow freezing or vitrification with different additional cryoprotectants as follows: dimethyl sulfoxide (DMSO), polyvinylpyrrolidone (PVP), combined DMSO and PVP (each at half the concentration of when used independently), or none (control). Cryopreserved ovarian tissues were evaluated by neutral red staining, histology, and xenotransplantation assays. Among cryopreservation conditions tested, vitrification with combined DMSO and PVP significantly improved the maintenance of follicular morphology compared to that in control. Furthermore, ovarian tissues vitrified using this condition maintained follicle morphology and developmental capacity 9 weeks after grafting, as shown by an increased percentage of primary and secondary follicles and a significant decrease in the transition stage from primordial to primary stage follicles 5 and 9 weeks after grafting. In contrast, slow freezing and control groups lost intact follicles by 5 weeks after grafting. The described cryopreservation techniques, which preserve canine follicle development, will build the foundation of ovarian tissue cryopreservation to preserve female fertility in wild canids.

## Introduction

Many animal species are threatened and endangered mainly due to habitat loss and direct human activities. Some others are considered genetically valuable based on their limited habitat or inbreeding issues associated with captive environments. In this context, gamete preservation has an important role in the conservation of such animals^1,2^. Cryopreservation of sperm has become a powerful tool to preserve male fertility and has been successfully applied to obtain offspring for many species when combined with artificial insemination (AI) or *in vitro* fertilization (IVF)^3^. In contrast, the preservation of female fertility is still challenging and has been associated with limited success^2^. This is due to difficulties in accessing the low numbers of adequate oocytes for oocyte cryopreservation or IVF, as well as the low success rate of oocyte cryopreservation. In addition, it is common for wild animals to die prior to reaching reproductive maturity or during the oestrus cycle, and it is difficult to obtain fertilizable oocytes, which is required for cryopreservation.

Alternatively, the cryopreservation and transplantation of ovarian cortical tissue have become a promising technique for the preservation of female fertility^4^. Within the ovarian cortex, there is an abundance of small preantral follicles, and in particular primordial follicles. The majority of oocytes from these primordial follicles never fully develop and thus never produce a viable, fertilizable oocyte^5^. The ability to preserve the pool of these small follicles and grow them to a mature stage with a competent oocyte would be enormously beneficial for preserving female fertility in such animals, regardless of age or reproductive timing. In addition, the survival rate of primordial follicles within the ovarian cortex after freezing is considered to be better than that of single matured oocytes due to their small size and arrested developmental stage at prophase of meiosis I^6^.

The ovarian tissue cryopreservation technique has been applied in medical fields to restore fertility in young women suffering from premature ovarian failure due to cancer treatment, genetic disorders, or other specific diseases^4,7^. To date, many healthy children have been born after the orthotopic transplantation of cryopreserved ovaries^4,7^. There are two methods for the cryopreservation of ovarian tissue, namely slow freezing and vitrification. Slow freezing is the most commonly employed cryopreservation technique for ovarian tissue cryopreservation^4,7^. This process is associated with very little toxicity and osmotic damage due to the use of low concentrations of cryoprotectants. However, slow freezing has several disadvantages including ice crystal formation and the use of an expensive programmed freezer^4,8^. Alternatively, vitrification is another cryopreservation method that involves the use of large concentrations of multiple cryoprotectants that can prevent the formation of ice crystals^8^. This method is a simple and low-cost technique because of the direct plunging/storing in liquid nitrogen and the fact that it does not require specific freezing machines. Thus, the simplicity and inexpensiveness of vitrification is particularly attractive to the field of wildlife biology, because it allows for the implementation by trained staff in any laboratory and it facilitates the accumulation of large numbers of samples for wild animals for future genome resource. Furthermore, needle immersion vitrification is a promising method of ovarian tissue vitrification^9^. This technique is attractive for use in conservation biology not only because of the ease of handling, but also because it provides uniform exposure to cryoprotectants and an increased cooling rate by direct cooling in liquid nitrogen^9^.

Although ovarian tissue vitrification can lead to pregnancy and live births in humans^10,11^, there are concerns about the relatively high concentration of cryoprotectants such as dimethyl sulfoxide (DMSO). The exposure of oocytes to high concentrations of cryoprotectants is known to damage these via both cytotoxic and osmotic effects^8,12^. Polyvinylpyrrolidone (PVP) is a synthetic polymer that has been used as a non-permeating cryoprotectant for vitrification^13^, and has shown promising results for different cells and tissues including oocytes^14–16^ and ovarian tissues^17–19^. However, PVP is not widely used for cryopreservation, and its impact on primordial follicles within ovarian tissues is unclear.

The domestic dog is a good model to study the conservation of wild canid species, as many of the 35 canid taxa are listed on the IUCN red list (International Union for Conservation Nature, 2018) in high categories with one species listed as critically endangered, four species listed as endangered, and five species listed as near threatened. There have been several studies involving the cryopreservation of canine ovarian tissue^20–26^, suggesting the possibility to cryopreserve these tissues in such species. Recently, Jivago *et al*. compared the techniques of slow freezing and vitrification in canine ovarian tissues and showed that vitrification was more effective than slow freezing in preserving the ultrastructure of primordial and primary follicles^20^. However, there is little information available regarding the specifics of primordial follicle developmental capacity with respect to different cryopreservation conditions or methods for ovarian tissue cryopreservation, and to our knowledge, no reports have been published on the influence of low-toxic cryoprotectants on canine ovarian tissues. Therefore, we conducted a comparative study on needle immersion vitrification using different cryoprotectants, including PVP, *vs*. slow freezing, with subsequent xenotransplantation assays, to determine the appropriate cryopreservation methods to optimally maintain primordial follicles within the ovarian tissues. The overall goal of the present study was to understand appropriate cryopreservation conditions for ovarian tissues that can sustain canine primordial follicle survival and developmental capacity.

## Results

### Study1: Effect of different cryopreservation conditions on follicle maintenance in canine ovarian tissues

**Figure 1:**
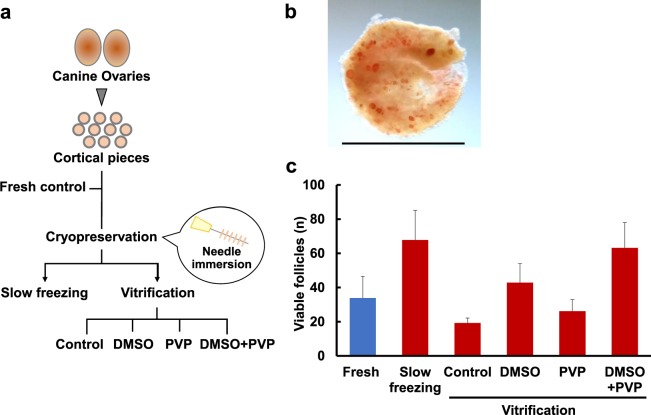
Effect of different cryopreservation conditions on follicle viability within ovarian tissues evaluated by neutral red (NR). (**a**) Overview of Study 1 comparing the effect of different cryopreservation conditions on follicle viability using canine ovarian tissues (n = 5 animals). 24–36 cortical pieces were divided into four to six pieces to allow direct comparisons of the five cryopreservation conditions and fresh tissues. Half of them used for the experimental conditions (i.e. two to three pieces/dog/group) were assessed for NR staining and another half were used for histological analysis. Control: vitrification with no additional cryoprotectant; DMSO: dimethyl sulfoxide; PVP: polyvinylpyrrolidone. (**b**) Photograph of ovarian cortical pieces after vitrification with DMSO + PVP stained with NR. Red points indicate viable ovarian follicles stained with NR. Bar = 2 mm. (**c**) Mean number (± SEM) of viable follicle stained by NR in samples of the same size (2 mm diameter) from the dog ovarian cortex before (fresh) and after cryopreservation with different cryopreservation conditions (slow freezing, control, DMSO, PVP, and DMSO + PVP).

We first investigated the effect of different cryopreservation methods (vitrification *vs*. slow freezing) and cryoprotectants on follicle viability and morphological maintenance. Based on many attempts using different cryoprotectants for vitrification, Mouttham and Comizzoli found that a combination of fetal bovine serum (FBS), sucrose, ethylene glycol (EG), and DMSO was effective to vitrify feline ovarian tissues^27^. The use of same combination of cryoprotectants with different concentrations was also successfully vitrified canine GV oocytes^28^. In addition to using this vitrification solution for canine ovarian tissues, we selected PVP, instead of DMSO, a combination of DMSO and PVP (each at half the concentrations of when used independently), and no additional agents (control) as alternative cryoprotectant conditions for vitrification (Fig. 1a). The viability of immature follicles within ovarian tissues was evaluated by neutral red (NR) staining^26^. All ovarian tissues before and after cryopreservation showed positively-stained follicles, which were considered viable follicles (Fig. 1b). The estimated number of viable follicles did not differ between fresh (33.9 ± 12.7) and cryopreserved–warmed tissues and among different cryopreservation treatments (*P* = 0.09; Fig. 1c). However, among treatments, there tended to be more NR-positive follicles in tissues vitrified with DMSO + PVP as cryoprotectants (63.2 ± 14.9), as well as in tissues frozen by slow freezing (67.8 ± 17.3), compared to those with other cryopreservation conditions (control, 19.3 ± 2.9; DMSO, 42.9 ± 11.1; PVP, 26.1 ± 6.8; Fig. 1c).

**Figure 2:**
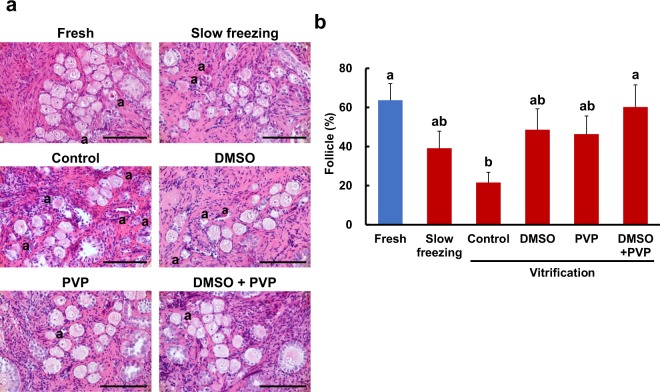
Effect of different cryopreservation conditions on follicle morphology within the ovarian tissue. (**a**) Histomicrographs of ovarian tissue before (fresh) and after cryopreservation with different cryopreservation conditions (slow freezing, vitrification with no additional cryoprotectant [control], dimethyl sulfoxide [DMSO], polyvinylpyrrolidone [PVP], and DMSO + PVP). “a” indicates the abnormal follicles. Bar = 50 µm. (**b**) Mean (± SEM) percentages of morphologically-normal follicles in ovarian tissue before (fresh) and after cryopreservation with different cryopreservation conditions (slow freezing, vitrification with no additional cryoprotectant [control], DMSO, PVP, and DMSO + PVP). Different letters indicate statistically significant differences (*P* < 0.05) in the percentages of normal follicles.

Trends in follicle maintenance evaluated by NR staining and histological analysis were similar, but histological analysis clearly revealed that cryopreservation conditions affected the normal morphology of ovarian follicles. Specifically, the percentage of morphologically normal follicles increased (*P* = 0.007) after vitrification with DMSO + PVP (60.2 ± 8.6%) compared to that of control conditions (21.6 ± 3.9%), and was comparable to that of fresh tissues (63.7 ± 10.2; Fig. 2a,b). The percent normality for other cryopreservation conditions were intermediate (slow freezing, 39.1 ± 5.1%; DMSO, 48.6 ± 8.3%; PVP, 46.3 ± 3.5%) and did not differ from those of control conditions (Fig. 2b).

Among donors, histology revealed no differences in the proportion of follicular stage. Further, there was no influence (*P* > 0.05) of cryopreservation treatments in follicular distribution, and thus each treatment had no stage-specific effect on follicular maintenance (Table 1). In fresh cortices, approximately 50% or more follicles were classified as primordial stage (56.1 ± 7.7%), whereas the rest were a transition from primordial to primary (18.9 ± 5.6%) and primary stages (15.5 ± 4.0%); follicles in secondary stages were minimal (9.4 ± 3.7%). This proportion did not change in cortical tissues after slow freezing or vitrification regardless of the use of different cryoprotectants (*P* = 0.82).

**Table 1:** Effect of different cryopreservation conditions on follicle distribution.

	Fresh	Slow freezing	Vitrification			
			Control	DMSO	PVP	DMSO + PVP
Primordial	56.1 ± 7.7	56.2 ± 12.2	56.1 ± 9.9	57.5 ± 8.3	55.8 ± 7.6	70.6 ± 6.2
Transition	18.9 ± 5.6	17.5 ± 1.5	20.2 ± 8.1	23.0 ± 7.9	26.7 ± 8.8	16.1 ± 5.7
Primary	15.5 ± 4.0	11.0 ± 4.5	12.8 ± 0.9	9.4 ± 1.5	8.7 ± 2.4	6.2 ± 1.5
Secondary	9.4 ± 3.7	15.3 ± 7.0	10.9 ± 5.7	10.1 ± 2.4	8.5 ± 3.3	7.1 ± 1.8

Mean (± SEM) percentages of follicle distribution across each stage (primordial, transition from primordial to primary, primary, and secondary) for ovarian tissue before (fresh) and after cryopreservation with different cryopreservation conditions (slow freezing, vitrification with no additional cryoprotectant [control], dimethyl sulfoxide [DMSO], polyvinylpyrrolidone [PVP] and DMSO + PVP).

### Study 2: Effect of ovarian tissue cryopreservation on immature follicles after xenotransplantation into immunodeficient rats

To understand how cryopreservation conditions might affect follicular viability and development *in vivo*, we performed xenotransplantation of cryopreserved ovarian tissues into immunodeficient rats. Based on the results of Study 1, three cryopreservation conditions, slow freezing, vitrification with a combination of DMSO and PVP, as well as vitrification without an additional cryoprotectant (control), were selected (n = 4–5/group).

Our histological observation confirmed that there were no differences on the proportions of morphologically normal follicles and their distribution from primordial to secondary stage before xenotransplantation among donors in each cryopreservation condition (data not shown).

**Figure 3:**
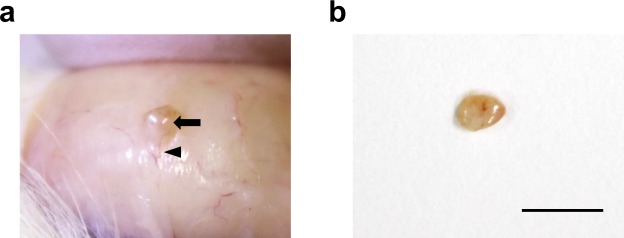
Gross morphology of vitrified ovarian tissue after xenotransplantation into immunodeficient rats. (**a**) Grafted tissues in the rat skin after vitrification and 5 weeks of grafting (arrow). Vascularization between skin and attached grafts was observed (arrowhead). (**b**) Collected grafts from rat skin after vitrification and 5 weeks of grafting. Bar = 5 mm.

**Figure 4:**
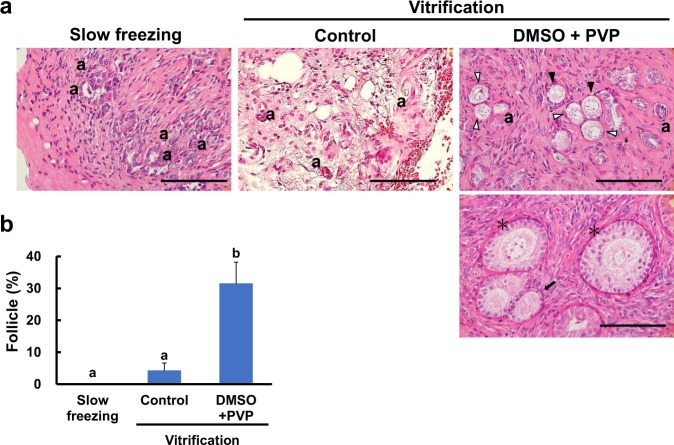
Effect of different cryopreservation conditions on follicle morphology after xenotransplantation into immunodeficient rats. (**a**) Histomicrographs of ovarian tissue cryopreserved by slow freezing or vitrification (control or dimethyl sulfoxide and polyvinylpyrrolidone [DMSO + PVP]) after 5 weeks of grafting. White arrowheads indicate primordial follicles; black arrowheads indicate the transition stage of primordial to primary follicles; arrows indicate primary follicles; asterisks indicate secondary follicles, “a” indicates the abnormal follicles. Bar = 50 μm. (**b**) Mean (± SEM) percentages of morphologically-normal follicles in ovarian tissue cryopreserved by slow freezing or vitrification (control or DMSO + PVP) after 5 weeks of grafting. Different letters indicate statistically significant differences (*P* < 0.05).

All ovarian tissue grafts were successfully recovered (100% tissue graft survival) after 5 weeks of grafting regardless of the cryopreservation condition. Most of the grafted cortical pieces were combined into one or two tissues, of approximately 4 mm^2^, and attached tightly to the rat skin, with apparent vascular formation (Fig. 3a,b). Histological observations of grafted tissues revealed the presence of structurally intact follicles, from primordial to secondary stages, with visually healthy stromal cells only in tissues vitrified with DMSO + PVP, after 5 weeks of grafting (Fig. 4a,b). Specifically, the tissues vitrified with DMSO + PVP retained 31.4 ± 6.7% of intact follicles. This value was significantly higher (*P* = 0.001) than that in control conditions (4.2 ± 2.4%) and in the slow freezing group, which had no healthy follicles, with complete disruption of oocyte nuclei, after 5 weeks of grafting (0%; Fig. 4a,b). The ovarian stroma in the slow freezing and control groups was also damaged or degenerate compared to that in the grafted tissue vitrified with DMSO + PVP (Fig. 4a).

**Figure 5:**
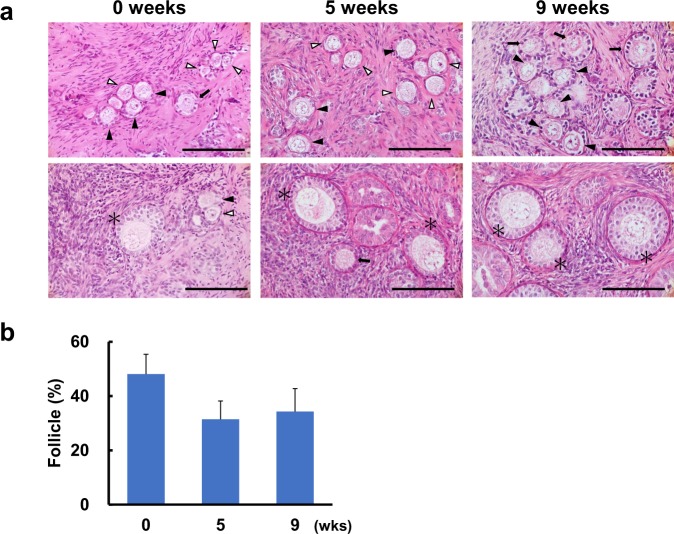
Effect of xenotransplantation on follicular morphology in vitrified ovarian tissues. (**a**) Histomicrographs of ovarian tissues vitrified with dimethyl sulfoxide and polyvinylpyrrolidone (DMSO + PVP) at 0, 5 and 9 weeks of grafting. White arrowheads indicate primordial follicles; black arrowheads indicate the transition stage of primordial to primary follicles; arrows indicate primary follicles; asterisks indicate secondary follicles. Bar = 50 μm. (**b**) Mean (± SEM) percentages of morphologically-normal follicles in ovarian tissue vitrified with DMSO + PVP at 0, 5, and 9 weeks after grafting.

Since the tissues vitrified with DMSO + PVP retained structurally normal follicles after 5 weeks of grafting, the xenotransplantation period was extended by 4 weeks for this group (total of 9 weeks; n = 3). The grafts were recovered successfully from all three recipients after 9 weeks of grafting; tissue graft survival in tissue vitrified with DMSO + PVP was 100% throughout the 9-week grafting period (Fig. 5). Moreover, structurally intact follicles from primordial to secondary stages were observed with morphologically normal stromal cells after 5 and 9 weeks of grafting (Fig. 5a). In addition, the percentage of intact follicles in the vitrified tissues did not differ (*P* = 0.25) from the onset to the end of grafting in this group (0 week, 48.1 ± 7.3%; 5 weeks, 31.4 ± 6.7%; 9 weeks, 34.3 ± 8.4%; Fig. 5b).

Xenotransplantation affected follicle distribution in vitrified tissues, as there was a shift in the percentages of primordial to primary and secondary stage follicles after 5 and 9 weeks of grafting (Table 2). Before grafting, almost half of the structurally normal follicles within the vitrified–warmed ovarian tissues were of primordial follicle stage (46.9 ± 11.0%), with 27.4 ± 3.0% in transition stage from primordial to primary follicles, 20.1 ± 7.3% primary follicles, and very few secondary stage follicles (5.6 ± 1.2%). Thus, the majority (approximately 75%) of the total follicle population was primordial or in transition stage from primordial to primary follicles. However, the percentage of primordial follicles in the vitrified tissues grafts was relatively low (*P* = 0.38) after grafting (5 weeks, 24.6 ± 15.5%; 9 weeks, 16.9 ± 1.0%), and there was a decrease (*P* = 0.01) in the percentages of follicles in transition stage from primordial to primary follicles after grafting (5 weeks, 15.5 ± 2.7%; 9 weeks, 11.9 ± 1.7%) compared to those in fresh vitrified–warmed tissues (27.4 ± 3.0%). Subsequently, the percentage of primary (*P* = 0.40) and secondary follicles (*P* = 0.53) increased in vitrified tissues after grafting; 37.2 ± 1.7% and 43.7 ± 1.3% were categorized as primary follicles and 22.7 ± 13.7% and 27.5 ± 1.0% were secondary follicles in vitrified tissues after 5 and 9 weeks of grafting, respectively (Table 2). Hence, the population of advanced-stage follicles in primary and secondary stages was predominant (more than 70%) after 9 weeks of xenografting. Of note, no antral formation was observed in the grafted ovarian tissues.

**Table 2:** Effect of xenotransplantation on follicle distribution in vitrified ovarian tissues.

	0 weeks	5 weeks	9 weeks
Primordial	46.9 ± 11.0	24.6 ± 16.5	16.9 ± 1.0
Transition	27.4 ± 3.0^a^	15.5 ± 2.7^b^	11.9 ± 1.7^b^
Primary	20.1 ± 7.3	37.2 ± 18.9	43.7 ± 1.3
Secondary	5.6 ± 1.2	22.7 ± 13.7	27.5 ± 1.0

Mean (± SEM) percentages of follicle distribution across each stage (primordial, transition from primordial to primary, primary, secondary) for ovarian tissue vitrified with dimethyl sulfoxide and polyvinylpyrrolidone (DMSO + PVP) at 0, 5, and 9 weeks after grafting. Within rows, different letters indicate significant differences (*P* < 0.05) in the percentages of follicles of each stage.

## Discussion

Ovarian tissue cryopreservation is a promising technique to preserve female fertility, including that in immature individuals. Whereas slow freezing is a standard technique for human ovarian tissue preservation, vitrification has proven to be an alternative technique for producing human babies^10,11^. Follicle survival was also demonstrated after ovarian tissue cryopreservation using either of these techniques in other mammalian species including dogs^20,21–26^. However, the equality or superiority of these techniques is still unclear, and optimal cryopreservation conditions to preserve ovarian follicle capabilities in other species still need to be developed. Using a domestic dog as a model for the wild canids, this study was the first to validate the effectiveness of PVP as a low-toxic cryoprotectant for ovarian tissue vitrification, based on follicular morphology and development in this species. Specifically, the present study demonstrates that a vitrification method, combining the use of DMSO and PVP as cryoprotectants, can successfully maintain follicle viability within canine ovarian tissues. Furthermore, our xenotransplantation assay proved that primordial follicles within the vitrified ovarian tissues, under these conditions, developed into primary and secondary follicles. We also discovered for the first time that needle immersion vitrification was superior to the present slow freezing method for preserving follicle development in this species.

Vitrification requires high concentrations of cryoprotectants to protect the oocytes and cells from rapid cooling. Since common cryoprotectants can be toxic to oocytes^8,12^, the selection of proper agents and concentrations is key to reducing their toxicity during the development of successful vitrification protocols for ovarian tissue cryopreservation. Addition of polymers, such as PVP, prevents ice crystal formation in vitrification solution as a non-penetrating cryoprotectant^13^. In our current study, the use of PVP resulted in similar post-warm viability compared to that in fresh tissues as well as that with DMSO, suggesting that it could be as effective as DMSO for canine ovarian tissue vitrification. Furthermore, the highest survival rate of immature follicles was achieved with a combination of DMSO and PVP, which also preserved follicle development after xenotransplantation. Therefore, our study suggested that PVP could reduce the toxicity of DMSO without compromising vitrification, thus improving the preservation of immature follicles. The beneficial effect of PVP on ovarian tissue vitrification was also previously reported in human^19^ and macaques^17,18^. The study of human ovarian tissues showed that vitrification with PVP preserved morphological characteristics of ovarian follicles and stroma^19^. In cynomolgus ovarian tissue, vitrification with this polymer, as an alternative to DMSO, improved the morphology of preantral (secondary) follicles and oocyte mitochondria at an ultra-structural level^18^. Another group also showed that vitrification with PVP improved the *in vitro* survival and hormone production of secondary follicles following post-warm culture in macaque ovarian tissue^17^. The difference between these previous studies and our study is that the ovarian tissues utilized in our system were in a more immature state; specifically, more than 90% of follicles in fresh ovarian cortices were classified as primordial to primary stages. Therefore, our findings provide strong evidence that PVP is beneficial to support follicle/oocyte survival and function throughout the different developmental stages during ovarian tissue vitrification for various mammalian species. However, whereas our study clearly indicated the advantage of PVP as a cryoprotectant, the vitrification solution associated with the best outcome still contained DMSO (albeit a lower concentration). Because it is possible that the vitrification properties of these permeable and non-permeable cryoprotectants are different, it would be worthwhile to investigate their associated mechanisms and interactions during ovarian tissue cryopreservation in the future.

Xenotransplantation assays demonstrated that our vitrification protocol is suitable for maintaining the integrity and follicular growth capacity of the canine ovarian cortex. The ovarian tissues vitrified with DMSO and PVP were not altered in terms of the proportion of intact follicles before and after xenotransplantation, indicating that follicle viability was maintained using our vitrification process. Although we could not observe a significant difference, probably due to the marked variation (SEM) at 5 weeks of grafting, the sharp increase in secondary follicle density after 9 weeks of xenotransplantation suggested that follicle development into secondary follicles occurred in vitrified ovarian tissues. To date, the vitrification of canine ovarian tissues following xenotransplantation has been reported by only one group, who reported difficulties in maintaining follicular integrity^21,24,25^. Despite the observation of primary follicles, earlier studies demonstrated that the majority of ovarian follicles disappeared, with no development into secondary follicles, in vitrified canine ovarian tissues after 4 weeks of xenotransplantation^21,24,25^. Although we did not monitor changes in follicle number, the proportion of intact follicles was maintained and a shift from primordial to primary and secondary follicles occurred after 9 weeks of xenotransplantation, indicating the improved preservation of canine ovarian tissues. Different reported outcomes are probably due to the different vitrification protocols. The previously reported results were based on a vitrification solution consisting of 2 M DMSO, 1 M acetamide, and 3 M propylene glycol^21,24,25^; in contrast, we used FBS, sucrose, EG, DMSO, and PVP. Additionally, whereas ovarian tissues were cooled together with the vitrification solution in these studies, we applied a needle immersion vitrification method. In this system, the tissues held by the needle are vitrified after wiping away the vitrification solution, thereby maximize the cooling rate and reducing the potential toxicity and osmotic effects of the vitrification solution, based on the use of a minimal volume of cryoprotectants^9^. Wan *et al*. showed that both mouse and human ovarian tissues were better preserved at the histological and ultrastructural level by needle immersion vitrification, compared to when tissues were cooled together with a drop of vitrification solution^9^. Therefore, in addition to the use of different cryoprotectant cocktails, it is possible that needle immersion vitrification also enhanced the preservation of canine ovarian tissues. The lack of antral follicles 9 weeks after grafting is in accordance with results obtained after the xenotransplantation of canine ovarian tissue frozen by slow freezing using a program freezer^22^. In that study, even though antral follicles were observed 1 week after grafting, no antral follicles were detected after 8 and 16 weeks, indicating the lack of follicle development at this stage^22^. Moreover, to our knowledge, development to the antral stage, not only after cryopreservation, has not yet been reported after canine ovarian tissue xenotransplantation. To obtain antral follicles and fertilizable oocytes from cryopreserved canine ovarian tissues, further improvement of xenotransplantation protocols, and perhaps a combination with *in vitro* culture, is essential for future applications of female fertility preservation. Nevertheless, our vitrification protocol can serve as a useful cryopreservation method for canine ovarian tissues to preserve follicle integrity and developmental capacity, and future improvements to this system will be made.

We also validated the value of using xenograft assays to confirm follicular survival after ovarian tissue cryopreservation. It could be suggested that changes in follicular quality and surrounding stroma cannot be detected based on earlier histological analysis. This was especially notable in the slow freezing group, where xenografting revealed evidence of a loss of follicle integrity. The complete loss of intact follicles after xenotransplantation in slow freezing tissues was unexpected, because our viability and histological analysis showed that the proportion of intact follicles in cryopreserved ovarian tissues were similar between slow freezing and vitrification groups. The recent study in canine ovarian tissues showed that follicles frozen by slow freezing presented ultrastructural damage, while vitrified follicles were well preserved^20^. In the present study, it is also possible that follicular integrity was already affected in the ultrastructural level after cryopreservation, and thus led to the complete follicular loss after xenografting in the slow freezing group. In addition to the follicular loss, strong degeneration of stromal cells was also observed after grafting ovarian tissues from the slow freezing and control groups. It is well known that the survival and development of primordial follicles is mechanically and physically supported by the surrounding stroma^29^. Complex bidirectional signalling between the oocyte and the surrounding stroma activate follicle development, for example, by secreting growth factors such as Kit ligand^29^ and by adjusting the extracellular matrix through the regulation of matrix metalloproteinases^30^. A study on bovine ovarian tissue cryopreservation showed that the stroma appeared to be more vulnerable to cryoinjury than the primordial follicles^31^. The study in human ovarian tissues showed that the ovarian stroma was significantly better preserved after vitrification than after slow freezing^19^. In the current study, it is possible that the fitness of stromal cells in cryopreserved ovarian tissues was already affected after cryopreservation, and therefore lost its ability to support follicle survival after xenotransplantation in slow freezing and control groups. Our observation suggested that without knowing the long-term survival and developmental ability of ovarian follicles, it is difficult to assess the follicular maintenance of cryopreserved ovarian tissues. Whereas *in vitro* culture is another useful assay to understand the biological mechanisms that occur during follicle development and to assess cryopreservation protocols quickly^26^, the limitation, particularly in dogs, is that canine ovarian tissue is difficult to maintain for long term *in vitro*^32^. Therefore, future studies should continue to rely on both evaluative approaches.

Individual variation of donor animals needs to be considered when evaluating an influence of different cryopreservation conditions. Our histological examination showed that ovaries from all donors appeared morphologically similar including the proportion of follicular stage. Moreover, our preliminary experiments revealed that the outcome of xenotransplantation in each cryopreservation condition did not differ when each donor was solely used for any of single condition and when the donor was shared with different conditions. Therefore, although there was variation between donor animals in terms of age, breed and storage time of ovaries, an influence of animal variation on current studies was minimal.

In conclusion, needle immersion vitrification with PVP can morphologically and functionally preserve immature follicles from canine ovarian tissues. Our convenient vitrification method, with a relatively low-toxic vitrification solution and developed using the domestic dog, provide a promising cryopreservation protocol to retain follicular developmental capacity, which could be applied to wildlife, and especially canids. Moreover, the combination of successful ovarian tissue cryopreservation and future breakthroughs in the induction of complete folliculogenesis will offer significant advancements for the preservation of fertility in endangered species.

## Methods

### Chemicals

All chemicals were purchased from Sigma-Aldrich (USA) unless otherwise indicated.

### Animals

All animal care and procedures performed in this study conformed with the Guidelines for Animal Experiments of Kyoto University, and were approved by the Animal Research Committee of Kyoto University (Med Kyo 17558).

### Collection of canine ovarian cortices

Ovaries were from domestic dogs of known age (6 months to 4 years) that underwent routine ovariohysterectomies at local veterinary clinics. Upon excision of the reproductive tract, each ovary pair was removed, immersed in L-15 medium containing 10 mM HEPES, 100 μg/ml penicillin G sodium, and 100 μg/ml streptomycin sulfate^33,34^, and transported to the laboratory (at 4 °C) within 18–24 h of surgery. Corpora lutea were not observed in any ovaries.

Ovarian cortical slices (1-mm thick) were dissected from the ovarian surface of both side of ovaries in working medium of Hanks’ MEM (Thermo Fisher Scientific, USA) supplemented with 15 mM Hepes, 2 mM glutamax (Thermo Fisher Scientific), 100 μg/ml penicillin G sodium (Wako Pure Chemical Corporation, Japan), 100 μg/ml streptomycin sulfate (Wako Pure Chemical Corporation), and 1% BSA^33^. The large follicles were mechanically removed from cortical slices and tissues were sectioned in equal pieces with a 2-mm diameter biopsy punch (Kai Corporation, Japan) on a warming plate (37 °C). At least three pieces from each donor were utilized as the fresh control and the rest were cryopreserved separately under 5 different conditions in each donor as described below.

### Neutral red staining to estimate viable follicles in fresh and cryopreserved–warmed tissues

Follicle viability within ovarian tissues was evaluated before and after cryopreservation using NR staining. NR stain readily diffuses through the plasma membrane and concentrates in the lysosomes of viable cells^35,36^; this was previously found to be effective for monitoring the survival of intraovarian follicles within the ovarian cortex^37^. Two or three pieces of fresh or cryopreserved–warmed cortices were randomly selected and rinsed in working medium of Hank’s MEM supplemented with 2 mM glutamax, 100 μg/ml penicillin G sodium, 100 μg/ml streptomycin sulphate, 15 mM Hepes, and 0.1% BSA. Tissue pieces were transferred to a 4-well dish (Thermo Fisher Scientific) and incubated in working medium supplemented with 33 μg/ml of NR solution (2-amino-3 methyl-7-dimethyl-aminophenazoniumchloride) for 2 h at 38.5 °C. The number of red coloured follicles stained with NR was counted in each cortical piece by observing the inner side of cortex under a stereomicroscope (Nikon, Japan).

### Histological analysis and classification of follicular structure

Histological analysis and classification of follicle structure were performed as described previously with some modification^32,33^. Briefly, ovarian tissues were fixed in Bouin’s solution (Wako Pure Chemical Corporation), maintained at 4 °C overnight, dehydrated in a graded series (70–100%) of ethanol solutions, and embedded in paraffin. Serial sections (5-μm thick) from each cortical piece were cut and stained with haematoxylin and eosin (both Muto Pure Chemicals). Only follicles containing oocytes with a visible nucleus were assessed. For each ovarian piece, three sections, including the largest area at the centre and one piece before and another after, each at least 20 µm from the middle section, were evaluated by light microscopy (Nikon ECLIPE E600, Nikon; 400×).

All follicles within the cortical pieces were characterized as follows: ‘normal’, when the nucleus of the oocyte and the surrounding granulosa cells were structurally intact or ‘abnormal’, wherein the oocyte and/or granulosa cells contained a pyknotic, fragmented, or shrunken nucleus^32^. The percentages of morphologically normal follicles per section were calculated by dividing the number of normal follicles by the total number follicles evaluated multiplied by 100. Normal follicles were further classified as primordial (one layer of flattened granulosa cells around the oocyte), transitioning from primordial to primary (a single mixed layer of flattened and cuboidal granulosa cells), primary (a single layer of all cuboidal granulosa cells), or secondary (two or more layers of cuboidal granulosa cells)^33^. 

### Cryopreservation and warming procedure

The vitrification was performed using a needle immersion vitrification protocol described previously^27^ with some modification. Briefly, ovarian cortical pieces were threaded onto a 30 G needle (five to six pieces per needle; Tochigi Seiko, Japan) with space between each piece. The needles were first rinsed in Hank’s MEM supplemented with 20% FBS and immersed in equilibration solution consisting of 20% FBS and 7.5% EG in MEM supplemented with either no additional cryoprotectant (VF1; control), 7.5% DMSO (VF2), 2.5% PVP (average molecular weight 40000, VF3), or 3.75% DMSO and 1.25% PVP (VF4) for 10 min at 4 °C. The needles were subsequently transferred into vitrification solution consisting of 20% FBS, 15% EG, and 0.5 M sucrose in Hank’s MEM supplemented with either no additional cryoprotectant (VF1; control), 15% DMSO (VF2), 5% PVP (VF3), or 7.5% DMSO and 2.5% PVP (VF4) for 10 min at 4 °C. Then, after excess solution was quickly absorbed using a Kimwipe, the needles were directly plunged into liquid nitrogen, inserted into a cryovial (Watson, Japan) containing liquid nitrogen, and stored for at least 72 h.

For slow freezing, ovarian cortical pieces were also threaded with 30 G needle (five to six pieces/needle) to standardize tissue conditions among cryopreservation protocols. The needle was incubated with slow freezing solution consisting of 10% DMSO and 0.1 M sucrose in Hanks’ MEM for 15 min at 4 °C. After removal from slow freezing solution, the needles were inserted into a pre-cooled empty cryovial. Each cryovial was placed into a Nalgene Mr. Frosty freezing container (Thermo Fisher Scientific) with isopropyl alcohol (Wako Pure Chemical Corporation) to achieve a cooling rate of −1 °C min^−1^ cooling rate and then the freezing container was transferred to a −80 °C freezer. After at least 24 h, all cryovials were removed from the unit, immersed directly into liquid nitrogen and stored for at least 72 h.

For warming, needles holding tissue pieces were removed from the cryovial in liquid nitrogen and immediately transferred into a washing gradient solution. The vitrified tissues held by the needles were washed in gradient solution (Hank’s MEM with 20% FBS supplemented with 1, 0.5, 0.25, and 0 M sucrose) for 5 min at each step at 37 °C. For tissues frozen by slow freezing, the needles holding the tissue pieces were washed with the latter two solutions (Hank’s MEM with 20% FBS supplemented with 0.25 and 0 M sucrose) for 10 min at each step at 37 °C. The cortical pieces were then removed from the needles for further assessments.

### Xenotransplantation of cryopreserved ovarian tissues into immunodeficient rats and graft recovery

Female F344 SCID rats (supplied from NBRP, 6–10-weeks old; n = 8) were used as recipients for the xenotransplantation of cryopreserved canine ovarian tissues. Rat were housed (up to two per cage) in air-filtered, positive-pressure cages under specific pathogen-free conditions and had free access to water and food pellets. Animals were maintained in an air-conditioned (temperature, 24 ± 2 °C; humidity, 50 ± 10%) and light-controlled room (lights on from 07:00 to 19:00).

Bilateral ovariectomy was performed to eliminate the effects of endogenous gonadal hormones at least 1 week before grafting. Briefly, anaesthesia was maintained via the inhalation of 1–2% (v/v) isoflurane in oxygen and a dorsomedial incision was made on the dorsal skin along the spine and bilateral incisions were made in the dorsal body wall on each side of the spinal column to perform.

13 to 19 cortical pieces were cryopreserved with either of the following three different cryopreservation conditions: slow freezing, no additional cryoprotectant (control; VF1) and DMSO + PVP (VF4). Cryopreserved tissues were warmed as described, immersed in working medium, and transferred to the animal experiment facility within 30 min of thawing. Two sets of five to eight tissues recovered from different cryopreservation conditions were combined with 10 μl of Matrigel (Corning, USA) supplemented with 200 ng/ml of rat vascular endothelial growth factor (VEGF, Wako Pure Chemical Corporation) and 200 ng/ml of rat basic fibroblast growth factor (bFGF, Pepro Tech, USA), and incubated for 0.5 h at 37 °C for solidification. Anaesthetic was induced and maintained in the ovariectomised rat using isoflurane gas. Four or six, 1-cm long incisions were made in the rat skin over the back, and tissues with Matrigel were inserted into one of the incisions. Two sets of cryopreserved-warmed tissues from each replicate were transplanted into two rats separately for the different collection periods (5 and 9 weeks), and each rat harboured four or six grafts from different groups or replicates. After grafting, incisions were closed with skin staples (Becton, Dickinson and Company, USA). At 4 and 8 weeks after grafting (1 week before collection), recipient rats were injected with 75 IU/kg human chorionic gonadotropin (hCG; Teikokuzoki Co., Japan) intraperitoneally to promote follicle development. At 5 and 9 weeks after grafting, recipient rats were euthanized by a CO_2_ overdose and cervical dislocation and the grafts were excised from the dorsal skin incision and immediately fixed for histological evaluation.

## Experimental Designs

### Study 1: Effect of different cryopreservation conditions on follicle maintenance in canine ovarian tissues

The effect of different cryopreservation conditions on follicle maintenance was investigated using ovarian cortical pieces from 5 donors, each of which were cryopreserved with 5 different conditions. 24 to 36 cortical pieces combined from both ovaries was divided into four to six pieces and randomly allocated to six experimental groups as follows: fresh, slow freezing, VF1: no additional cryoprotectant (control), VF2, VF3, and VF4 (four to six cortical pieces/dog/group). From each of the six experimental groups, two to three cortical pieces were assessed for NR staining (n = 5 dogs) and another two to three pieces for histology (n = 4 of same 5 dogs of NR staining).

### Study 2: Effect of ovarian tissue cryopreservation on immature follicles after xenotransplantation into immunodeficient rats

Based on the results of Study 1, we performed xenotransplantation to investigate the effect of ovarian tissue cryopreservation by vitrification with DMSO + PVP (VF4) and slow freezing on follicle maintenance and developmental capacity *in vivo* (n = 5 donor dogs/group). Vitrification with no additional cryoprotectants (VF1) was the control (n = 4 donor dogs). In this study, we compared the results of xenotransplantation using different cryopreservation conditions performed on tissue obtained from either same or different animals. Five to eight cortical pieces in each experimental group were transplanted into two immunodeficient rats, each as described above (two sets of five to eight cortical pieces/donor/group). One set of grafts derived from each cryopreservation condition was collected after five weeks of grafting. Another set of grafted tissues vitrified with DMSO + PVP (VF4) from three of the same five donors was further maintained in immunodeficient rats and recovered nine weeks after grafting (n = 3). Collected grafts after five and nine weeks were processed for histological analysis as described above. Cryopreserved tissues from all donor dogs were also fixed and assessed for histological analysis immediately after warming (0 weeks of grafting, three cortical pieces/donor/group).

### Statistical analysis

Data are presented as means ± standard error of mean (SEM). A Shapiro-Wilk test was performed to evaluate the normality of the dataset, and a Bartlett test was used to confirm homogeneity of variances. Comparisons in follicle viability, morphology, and distribution among treatments were evaluated by analysis of variance (ANOVA) followed by a Turkey’s multiple comparison test. Differences were considered significant at P < 0.05 (GraphPad PRISM 7, GraphPad Software, USA).

## Data Availability

The datasets generated and/or analysed during this study are available from the corresponding author upon reasonable request.
